# Seroprevalence of hepatitis E in blood donors in Santiago, Chile: a monocentric cross-sectional study

**DOI:** 10.1016/j.ijregi.2026.100982

**Published:** 2026-08-22

**Authors:** Lorena Porte, Ian Batista Weitzel, Costanza Martínez-Valdebenito, Gabriel Pizarro, Verónica Bustamante, Thomas Weitzel

**Affiliations:** 1 Laboratorio Clínico, Clínica Alemana, Facultad de Medicina Clínica Alemana, Universidad del Desarrollo, Santiago, Chile; 2 Departamento de Enfermedades Infecciosas e Inmunología Pediátricas, Escuela de Medicina, Pontificia Universidad Católica de Chile, Santiago, Chile; 3 Banco de Sangre, Hospital Dipreca, Santiago, Chile; 4 Instituto de Ciencias e Innovación en Medicina, Facultad de Medicina Clínica Alemana, Universidad del Desarrollo, Santiago, Chile; 5 Institute of International Health, Charité Center for Global Health, Charité–Universitätsmedizin Berlin, Berlin, Germany

**Keywords:** Viral hepatitis, Hepatitis E virus, Epidemiology, Foodborne diseases, Emerging infectious diseases, South America

## Abstract

**Objectives:**

Hepatitis E virus (HEV) infection is emerging but under-recognized worldwide. However, the epidemiology and ecology of this infection in Chile are poorly understood. This study aimed to detect and analyze hepatitis E seroprevalence among blood donors at a private health care center in Santiago, Chile.

**Methods:**

Leftover sera were collected from 354 volunteer blood donors aged 18-65 years in July 2020. After recording sex, age, and municipality of residence, the specimens were anonymized and tested for anti-HEV immunoglobulin G antibodies using a commercial enzyme-linked immunosorbent assay kit (Wantai BioPharm). Municipalities were classified according to socioeconomic status as a proxy for income level. Statistical analyses were conducted using R software.

**Results:**

The overall seroprevalence was 15.3% (95% confidence interval, 11.9-19.4%). The prevalence increased significantly with age (50-65 years, 33.9%) and was higher among residents of lower-income municipalities than among those of higher-income municipalities (21.9% vs 11.7%). Multivariate analysis confirmed that age 50-65 years (odds ratio, 20.2) and residence in a lower-income municipality (odds ratio, 2.3) were independent predictors of HEV seropositivity.

**Conclusion:**

This study provides information on HEV exposure in adults in Santiago, Chile. Seroprevalence was highest in older age groups and among residents of lower-income municipalities. Further studies on HEV are needed to clarify its transmission cycle, circulating genotypes, and clinical relevance in Chile.

## Highlights

• Blood donors in Santiago, Chile, had a hepatitis E seroprevalence of 15.3%.
• Age 50-65 years was a strong predictor of hepatitis E seropositivity.
• Residents of lower-income municipalities had higher hepatitis E seropositivity than residents of higher-income municipalities.
• Further research on transmission cycles and clinical relevance of hepatitis E virus is needed.

## Introduction

The hepatitis E virus (HEV) is a non-enveloped, positive-sense, single-stranded ribonucleic acid virus belonging to the family *Hepeviridae,* genus *Paslahepevirus* [1]. HEV is among the leading causes of acute hepatitis worldwide and, according to the World Health Organization, is responsible for 5.4% of global disability-adjusted life years related to acute hepatitis [2,3]. Of the eight known HEV genotypes, four are most significant in human diseases (HEV-1 to HEV-4) [4]. HEV has at least two distinct epidemiological patterns depending on the predominant genotype and the economic and cultural conditions [5]. HEV-1 and HEV-2 mainly affect developing countries, where the virus is transmitted fecally or orally among humans, and poor sanitation can lead to large waterborne outbreaks [2,6]. In industrialized countries, zoonotic infections with HEV-3 and HEV-4 are predominant and are transmitted through the consumption of raw meat and meat-derived products from infected animals, particularly pigs, as well as wild boars, deer, and rabbits [2,6]. Depending on sociocultural habits, some regions in high-income countries such as Corsica in France are considered hyperendemic [7].

HEV infections are often oligosymptomatic; however, certain patient groups are at increased risk of severe manifestations [2]. HEV-1 and HEV-2 can cause severe acute infections in pregnant women, with mortality rates reaching up to 30%, whereas HEV-3 and HEV-4 may cause chronic infections, which in immunosuppressed patients can progress to liver cirrhosis [2,5,8]. Despite its clinical relevance, the global epidemiology of HEV remains incompletely defined [8]. The infection is under-recognized due to a lack of awareness among physicians. Furthermore, diagnostic tools are often not available, especially in resource-poor settings [8,9].

Data on HEV seroprevalence vary depending on the geographical region, study population, and performance of the applied immunoassays [10]. Blood donors are used as proxies to estimate HEV endemicity in a broader adult population [10]. Donor seroprevalence in Europe ranges from 4.7% in Scotland to 52.5% in southwestern France [10]; intermediate levels have been reported in studies from Spain (20.0%) [11] and Germany (29.5%) [12]. In Middle Eastern countries, blood donor seroprevalence ranges from 3.2% in Saudi Arabia to 28.8% in Egypt, with 20.7% reported in Qatar [13]. A recent systematic review in Southeast Asia reported a pooled HEV seroprevalence in blood donors of 11.8% [1].

In Latin America, hepatitis E is a neglected infection owing to limited laboratory infrastructure and clinical awareness, hindering surveillance studies [14,15]. A recent meta-analysis reported a pooled blood donor seroprevalence of 6.0% (95% confidence interval, 5.0-8.0%) in the region, with heterogeneous values ranging from 0.9% in northeastern Brazil to 30.1% in Santiago, Chile [16]. Its prevalence may vary significantly among countries. Among blood donors in Argentina, for example, seroprevalence ranged from 5.1% to 20.0% within five regions, with an overall value of 11.3% [17]. Data on HEV genotypes in Latin America are limited. Studies in Mexico have indicated the circulation of HEV-1 and HEV-3 in humans, whereas in Cuba, HEV-3 has been detected in humans and swine [16]. In several South American countries (Brazil, Argentina, Uruguay, Colombia, and Bolivia), HEV-3 was reported in human cases and/or samples from swine, pork products, and environmental matrices [15,18,19]. In Brazil and Argentina, zoonotic transmission of HEV-3 was supported by genetic analyses showing a close relationship between human and swine isolates [5,15], whereas in Bolivia, human HEV-3 strains differed from those of swine [15].

In Chile, hepatitis E infection is clinically under-recognized. Serological tests for HEV are available only in a few private laboratories; therefore, HEV testing is not incorporated into the diagnostic workup for cases of acute hepatitis. To date, only five cases of acute hepatitis E have been reported in Chile, all of which were diagnosed using single-sample serology [20,21]. In addition, a pregnant woman with fulminant hepatitis has been reported as a possible case of hepatitis E; however, diagnostic confirmation was not available [22]. Evidence of human HEV exposure in Chile is derived from a few small seroprevalence studies using distinct diagnostic tests. Blood donor surveys showed regional differences with a lower prevalence in southern Chile (8%) than in Santiago (31.1-32.6%) [23,24]. A comparative study from different South American countries showed that Chilean patients with chronic liver disease had a higher HEV seroprevalence (49.6%) than those from Argentina (4.5%), Brazil (21.7%), Colombia (6.9%), Ecuador (16.4%), and Peru (19.6%) [4]. A serosurvey of individuals with human immunodeficiency virus in Santiago revealed a seroprevalence of 10.4%, with an age-dependent increase from 0% in individuals born in the 1990s to 18% in those born before 1960 [25]. Due to the lack of studies and molecular diagnostic capacities, the circulating human HEV genotypes and modes of transmission in Chile are unknown.

The present study aimed to determine and analyze HEV seroprevalence among blood donors at a private healthcare center in Santiago, Chile.

## Materials and methods

**Figure 1:**
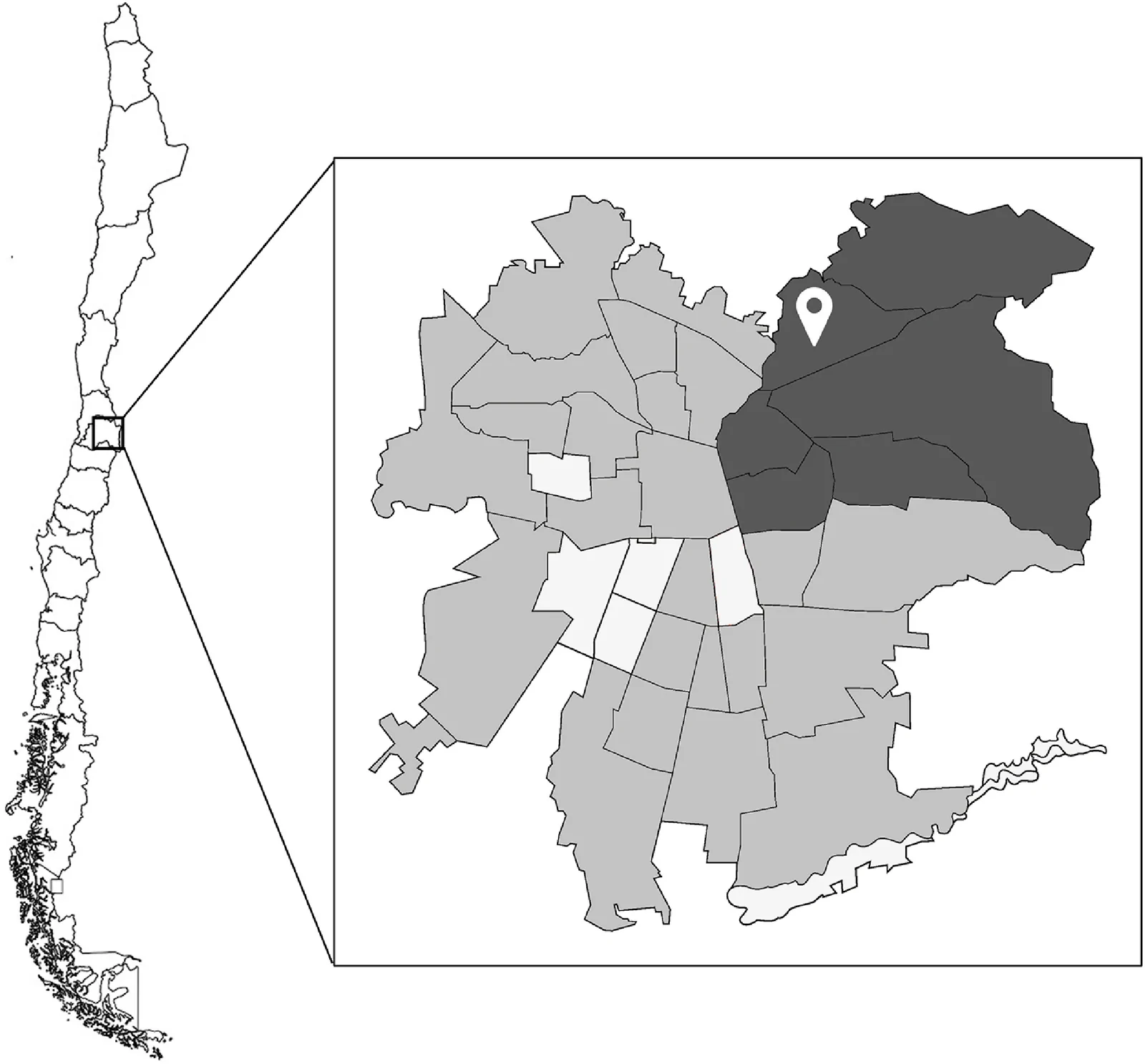
The city of Santiago is located within the Metropolitan Region in central Chile (left map). The right map shows municipalities of Greater Santiago. Blood donors resided in municipalities with higher socioeconomic status (dark gray shading) and lower socioeconomic status (light gray shading). The white pin shows the location of Clínica Alemana (Vitacura municipality). Maps: Wikimedia Commons under CC-BY-SA-4.0 license.

We conducted a retrospective observational study to estimate the seroprevalence of anti-HEV immunoglobulin G (IgG) antibodies in blood donors at Clínica Alemana, Chile. Clínica Alemana is a private tertiary health care center located in Vitacura, an upper-income municipality of Greater Santiago. Greater Santiago accounts for 36% of the country’s population (∼7 million people) (Figure 1) [26].

Leftover sera from volunteers who donated blood in July 2020 were aliquoted and frozen at -80°C until analysis in December 2020. Samples were tested for anti-HEV IgG antibodies using an enzyme-linked immunosorbent assay (ELISA; Wantai BioPharm, Beijing, China) on an automated platform (Immunomat, Virion/Serion, Würzburg, Germany), according to the manufacturer’s instructions. Briefly, the assay detects HEV-specific IgG antibodies after binding to recombinant HEV antigens and incubating them with horseradish peroxidase-conjugated anti-human IgG. The results were interpreted as recommended by the manufacturer, and the cutoff value was calculated as the mean optical density of the negative control plus 0.16. Samples with an optical density ratio ≥1.0 relative to the cutoff were classified as positive. Specimens with a ratio of 0.9-1.1 were retested for confirmation.

Samples were anonymized using a donation number provided by the Blood Bank Laboratory Information System (Hexa-Bank, Tharsis-it, Bogotá, Colombia). Basic demographic data, including sex, age, and municipality of residence, were collected. Residential municipalities were classified according to socioeconomic status (SES) as previously described [26]. Owing to the small sample size, we used two SES categories to analyze possible associations with HEV seroprevalence: higher-income municipalities (very high and high SES) and lower-income municipalities (middle, low, and very low SES) (Figure 1).

A sample size of 318 was determined using the Raosoft calculator (Raosoft Inc., www.raosoft.com/samplesize.html), with a 95% confidence level, 5% margin of error, and an assumed prevalence of 30%. Other statistical analyses were conducted using R software (version 4.4.0; https://www.r-project.org). The prevalence values were calculated with 95% confidence intervals. Further statistical analyses were performed using a univariate logistic regression model, a multivariate logistic regression model using age as a continuous variable (model 1), and a multivariate logistic regression model using age groups (model 2). Sex and municipality of residence were included as covariates. Statistical significance was assessed using Wald tests, with *P* < 0.05 considered statistically significant. A heatmap was created using GraphPad Prism version 10.6.1 (GraphPad Software, San Diego, CA, USA), and four age groups were used to better visualize age-dependent trends.

## Results

A total of 354 consecutive samples were analyzed. The median age of the donors was 35 years (range, 18-65 years; interquartile range, 27-45 years), and 54.8% were male (Table 1). Most donors (92.4%, n = 327) lived in metropolitan areas. Of these, 65.1% were from higher-income municipalities (high-income, n = 70; very high-income, n = 143) (Table 1, Figure 1). The overall HEV seroprevalence was 15.3%, with a higher rate in men (16.5%) than in women (13.8%). At 33.9%, the prevalence was higher in individuals aged 50-65 years than in those aged 30-49 years (16.8%) or <30 years (3.5%). The prevalence in lower-income municipalities was almost twice that of higher-income municipalities (21.9% vs 11.7%) (Table 1).

**Table 1:** Hepatitis E prevalence values by age groups, sex, and municipality of residence.

Variable	Category	*N*	HEV positive	Prevalence	95% CI
Age group (n = 354)	18-29 years	113	4	3.5%	1.4-8.8
	30-49 years	185	31	16.8%	12.1-22.8
	50-65 years	56	19	33.9%	22.9-47.0
Sex (n = 354)	Female	160	22	13.8%	9.3-19.9
	Male	194	32	16.5%	11.9-22.4
Municipality^a^ (n = 327)	Higher-income	213	25	11.7%	8.1-16.8
	Lower-income	114	25	21.9%	15.3-30.4
Total		354	54	15.3%	11.9-19.4

a Data were unavailable for 27 donors. Abbreviation: CI, confidence interval; HEV, hepatitis E virus.

**Figure 2:**
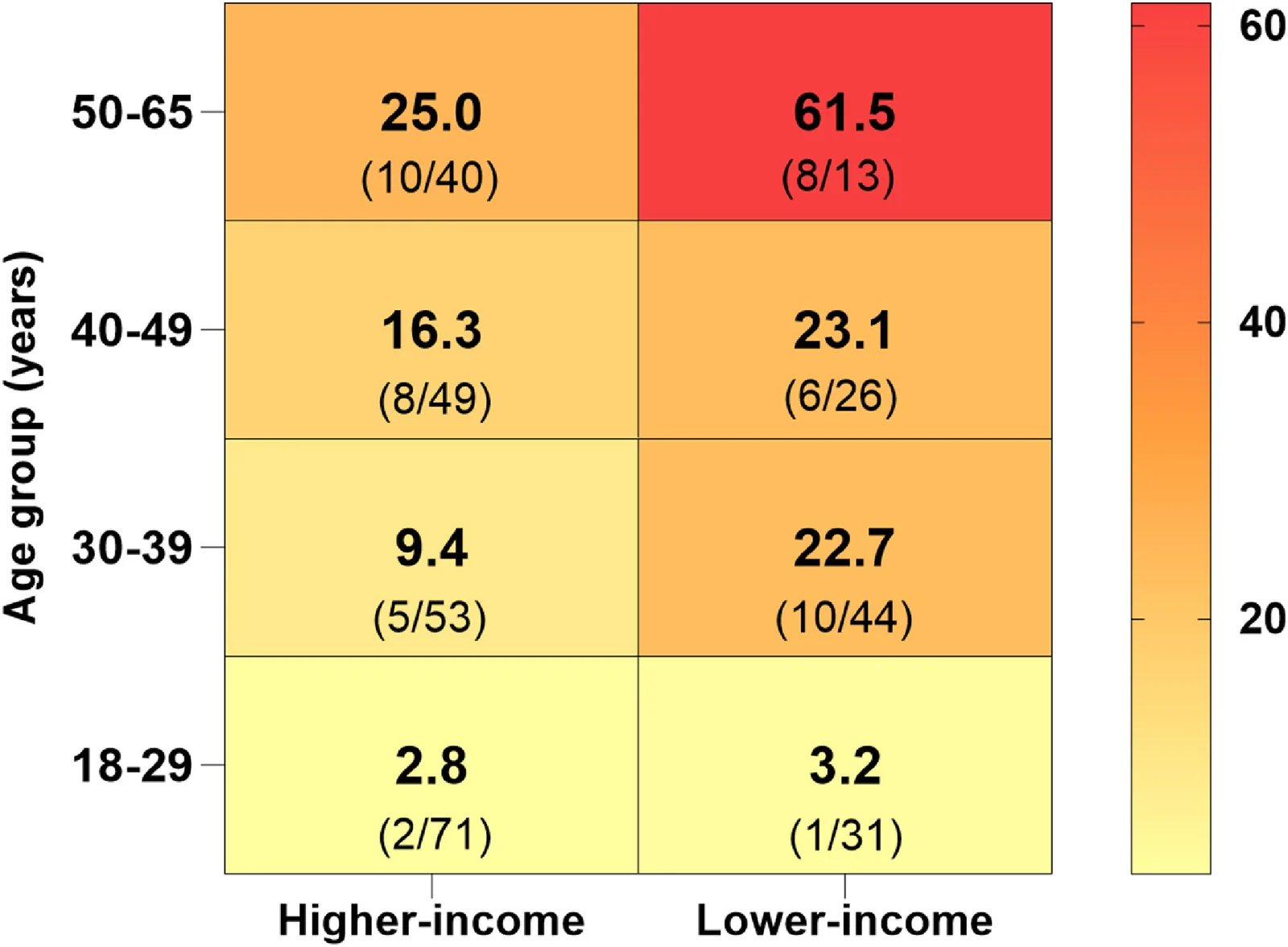
Heatmap of hepatitis E seroprevalence stratified by age groups and municipality of residence. Numbers are percentages (n/N).

Univariate analysis indicated that older age and residence in a lower-income municipality were associated with HEV seropositivity (Table 2). Multivariate analysis confirmed these findings, showing that individuals aged 50-65 years had 20.2 times the odds of HEV seropositivity compared with those in the youngest age group (<30 years) (Table 2). Individuals from lower-income municipalities had 2.3 times the odds of HEV seropositivity compared with those living in higher-income neighborhoods (Table 2). The highest hepatitis E seropositivity was observed in those aged 50 years or older living in lower-income municipalities (Figure 2).

**Table 2:** Univariate and multivariate analyses of HEV seropositivity by age, sex, and municipality of residence.

Variable	Univariate	*P*	Multivariate (model 1)^a^	*P*	Multivariate (model 2)^a^	*P*
Age (years)	1.07 (1.04-1.10)	<0.00001	1.08 (1.05-1.11)	<0.00001	–	–
Age group						
18-29 years	1.00	–	–	–	1.00	
30-49 years	6.36 (2.45-21.74)	0.0006	–	–	7.38 (2.54-31.37)	0.001
50-65 years	15.14 (5.32-54.70)	<0.00001	–	–	20.15 (6.31-90.40)	<0.00001
Sex: female	0.80 (0.45-1.40)	0.45	0.98 (0.52-1.82)	0.95	0.96 (0.51-1.79)	0.91
Municipality: lower-income	1.87 (1.04-3.38)	0.03	2.34 (1.24-4.46)	0.008	2.07 (1.09-3.93)	0.02

Values are odds ratios (95% confidence intervals).

a Complete-case analysis (n = 327). Abbreviation: HEV, hepatitis E virus.

## Discussion

The interpretation of HEV seroprevalence studies is affected by the lack of a serological gold standard [10]. The diagnostic performance of commercial ELISAs differs, leading to significant variations in the results under similar settings [2,16]. The assay used in the present study (Wantai BioPharm) is known for its high sensitivity compared with other tests [2]. For example, a study of blood donors in Spain showed a prevalence of 20%, as tested by Wantai, compared with 11%, as tested by another ELISA (Mikrogen) [11]. This difference is likely related to the ability to detect low HEV IgG titers resulting from exposure in the distant past [12].

The overall HEV seroprevalence of 15.3% was similar to that reported in blood donors in Argentina (15.2%) using the same assay [10]. Argentinian studies of donors using other assays showed heterogeneous seroprevalence values, for example, 3.5% in Córdoba (DiaPro assay) [27], 9.2% in Tucumán (in-house assay) [9], and 5.1-20.0% in a survey including five regions (DiaPro assay) [17]. Data from blood donors in Chile revealed a seroprevalence of 30.1% in Santiago in 2014 [23] and 6.1-18.8% in three cities in southern Chile in 1997 [24]. These values are difficult to compare because of the diversity of the techniques used. A decrease in seroprevalence over time in the city of Santiago (30.1% in 2014 vs 15.2% in 2020) could be associated with a reduced risk of fecal-oral infections, similar to hepatitis A and typhoid fever, which declined significantly after public health interventions during the cholera epidemic in the 1990s [28].

HEV seroprevalence in the donor population increases with age. Most probably, this represents a cohort effect, as older age groups were exposed to inferior hygienic conditions in Chile during earlier decades, combined with the long-term persistence of anti-HEV IgG antibodies [29]. Cumulative lifetime exposure effects have also been proposed for blood donors in Argentina and other industrialized nations [10,17].

Our analysis showed a significantly higher seroprevalence in residents of lower-income municipalities than in those of higher-income municipalities, particularly in the 50-65-year age group (61.5% vs 25.0%). An association among hepatitis E, national economic development, and income level has been described previously [2,8]. However, studies analyzing income-associated differences within the same country are scarce. In our study, the higher HEV seroprevalence in individuals from lower-income municipalities in Santiago could be associated with a higher risk of exposure to contaminated food or water during their lifetime. The coverage of piped drinking water and wastewater treatment has been near universal in Santiago since the 1990s [28]. However, higher levels of food insecurity in populations with lower SES are a continuing problem in Chile [30]. This is consistent with the high prevalence reported in public hospitals in Santiago, which serve a larger proportion of patients from lower-income neighborhoods [23,31]. Exposure to zoonotic genotypes is also possible through meat products, as HEV antibodies detected in 7-9.5% of pigs in central Chile suggest a reservoir in swine [32]. Further studies are necessary to clarify the endemicity of the different HEV genotypes and transmission cycles in Chile. As recommended by the World Health Organization and recently implemented in Argentina, environmental surveillance studies using wastewater are useful tools for unraveling the epidemiology of HEV [33].

The clinical significance of hepatitis E in Chile remains unresolved. Despite the existing seroprevalence, only five clinical cases of acute hepatitis E have been reported to date, all of which were diagnosed over two decades ago by single-sample serological HEV testing. One patient had a positive hepatitis E IgM result, three patients were IgG positive, and one patient had a positive unspecified experimental ELISA result [20,21]. Fulminant hepatitis in a pregnant woman was reported as a possible hepatitis E case, but without diagnostic confirmation [22]. The absence of further cases in Chile likely resulted from limited clinical awareness and a lack of testing infrastructure [8,22]. As in many other countries, infection is not a public health priority and is, therefore, not notifiable.

This study has some limitations. Our cohort was not designed to provide a national prevalence estimate; therefore, further studies with larger sample sizes are necessary to determine the prevalence over a wider geographical range in Chile. Our hospital mostly serves the higher-income sector of Santiago; thus, this study may have underestimated the prevalence in the general population. Specimens were collected by convenience sampling and derived from blood donors with a limited age range (18-65 years). Socioeconomic classification was based solely on the municipality of residence, without concrete information about the participants’ income and living conditions. Finally, information on the possible risk factors for HEV exposure was not available because of the retrospective study design.

In conclusion, this study provides updated epidemiological information on HEV exposure in Santiago, Chile. The study showed that the seroprevalence was significantly higher in older age groups and among residents of lower-income municipalities. Further research on HEV is warranted to understand its transmission cycle, circulating genotypes, and clinical relevance in Chile.

## CRediT authorship contribution statement

**Lorena Porte:** Data curation, Formal analysis, Investigation, Resources, Validation, Writing – original draft, Writing – review & editing, Project administration, Visualization. **Ian Batista Weitzel:** Data curation, Formal analysis, Investigation, Writing – original draft, Writing – review & editing. **Costanza Martínez-Valdebenito:** Formal analysis, Writing – review & editing. **Gabriel Pizarro:** Investigation, Supervision, Writing – review & editing, Data curation. **Verónica Bustamante:** Conceptualization, Investigation, Methodology, Project administration, Supervision, Writing – review & editing. **Thomas Weitzel:** Conceptualization, Formal analysis, Methodology, Project administration, Resources, Supervision, Validation, Writing – review & editing.

## Declaration of competing interest

The authors have no competing interests to declare.
